# LONELINESS AND OLDER ADULTS: RESULTS FROM A COMMUNITY-BASED INTERVENTION

**DOI:** 10.1093/geroni/igad104.1380

**Published:** 2023-12-21

**Authors:** Catherine Macomber, Paul Freddolino

**Affiliations:** Saginaw Valley State University, University Center, Michigan, United States; Michigan State University, East Lansing, Michigan, United States

## Abstract

The number of older adults identifying themselves as lonely has increased during the last two years as they have struggled with the impact of the pandemic on their perceptions of relationships with others. Recent research has shown that the negative health implications of loneliness are similar to those found with smoking or obesity. Most current interventions continue with past practice by adding people to engage with the older adult, although research suggests that while this intervention addresses social isolation, it does not address loneliness directly. Information regarding the development and implementation of a loneliness intervention with older adults based on the Health Beliefs Model in one rural mid-west county will be presented. Participants were recipients of home-delivered meals and had volunteered for a project that provided peer-to-peer instruction to improve digital literacy. In addition to technology training sessions, participants received a weekly contact from a separate volunteer as a friendly ‘check-in.’ Beginning in the fourth week, brief 5-10 minute coaching sessions about loneliness were included in these weekly contacts. The presentation will include details of the loneliness content, and describe training and support provided to the volunteers. Interviews were conducted with participants at baseline, approximately three months, and at the end of contact with each participant. Results showed no statistically significant change in loneliness, but participants reported they saw potential usefulness for the content with family and friends and potentially themselves in the future. They also provided helpful suggestions for revision. Plans for revision will be presented.

